# Rural-urban difference in meeting the need for healthcare and food among older adults: evidence from India

**DOI:** 10.1186/s12889-023-16126-4

**Published:** 2023-06-26

**Authors:** Jyoti Das, Sampurna Kundu, Babul Hossain

**Affiliations:** 1 International Institute for Population Sciences (IIPS), Mumbai, 400088 India; 2 Jawaharlal Nehru University (JNU), New Delhi, 110067 India

**Keywords:** Food and healthcare, Unmet need, Rural-urban gap, Longitudinal and Ageing Survey of India, India

## Abstract

**Background:**

Due to changes in demographic and epidemiological scenarios, and the gradual increase in the older population, India is yet to prepare for rising nutrition and health-related issues among older adults in the coming decades. While the process of ageing and its associated aspect has been found to have an urban-rural divide. Thus, this study examines rural/urban differences in unmet needs for food and healthcare among Indian older adults.

**Methods:**

A sample of 31,464 older adults aged 60 years and above were considered in the study from the Longitudinal and Ageing Survey of India (LASI). The bivariate analysis was done using the sampling weights. Logistic regression and decomposition analysis was used to explain the rural-urban gap in the unmet needs for food and healthcare among Indian older adults.

**Results:**

Rural older adults were more vulnerable to meeting the need for health and food than their urban counterparts. While factors that contributed majorly to the difference in unmet need for food between urban and rural were education (34.98%), social group (6.58%), living arrangements (3.34%) and monthly per capita expenditure (MPCE) (2.84%). Similarly, for the unmet need for health, the factors that contributed the most to the rural-urban gap are education (28.2%), household size (2.32%), and MPCE (1.27%).

**Conclusion:**

The study indicates more vulnerability among rural older adults than compared to urban older individuals. The targeted policy-level efforts should be initiated considering the economic and residential vulnerability identified in the study. There is a need for primary care services that can provide targeted help to older adults in rural communities.

## Introduction

Global mortality profile improved in stages; better quality of water, food, sanitation, housing and lifestyle, vaccination against infectious disease, antibiotics, and improved medical treatment first led to a decrease in infant mortality, followed by an increase in life expectancy [1, 2]. This longevity revolution is the most important upcoming demographic trend [3]. Globally, the number of older adults and their share of the population is increasing in every nation [4]. From 2019 to 2050, the senior population (defined as those aged 60 or over) is projected to expand from 900 million to 2 billion, with considerable increases in the number of people surviving to age 80 and beyond [5]. Although the ageing process began in industrialized nations, low- and middle-income countries (LMICs) are undergoing considerable transformation; by 2050, LMICs are projected to be home to two-thirds of the world’s older individuals. Despite the global rise in life expectancy, it has not resulted in an increase in healthy life expectancy in all areas [6]. In 2019, the healthy life expectancy at birth was 68 years in Europe, 62 years in Southeast Asia, and 58 years in Africa, according to World Health Statistics [7].

Considering most populated regions like Southeast Asia, the proportion of older adults are less than 10% of the total population. However, it becomes implausible when considering the size of the ageing population [8]. With the change in the demographic structure of a country, the need for modification in different aspects of the community also emerges [9]. These structural changes have affected the general lifestyle and achieving quality of life, which includes both consumption or/and availability of food and healthcare needs [10].

Brown et al. (2010) put forward that a healthy diet can minimize the effect of various physiological conditions on the ageing population [11]. While at a global level suffering more from insufficient food availability than any other age group population has been observed among older adults [12]. The causes behind this phenomenon put forward in various research were low income, immobility, fragile health, and social discrimination [12–14]. According to Food & Agriculture Org Report (2021), globally, around 2.37 billion population did not have access to adequate food in 2020; with available statistics from developed countries, 1 in every 15 older adults was vulnerable regarding sufficient food [15].

Adding to the vulnerabilities of older adults, healthcare needs have emerged to be a greater concern. The major health concerns faced by the ageing population are the incidence of chronic diseases and disabilities, which further makes them subject to increased long-term healthcare [16]. Moreover, failing to acquire required or delayed healthcare may aggravate existing health conditions and result in fatal conditions [17]. To meet global healthcare needs, Universal Health Coverage has been introduced and aspired by every country; however, instead of such initiatives, half of the world’s population suffers from an unmet need for healthcare access [18, 19]. Being a rapidly growing population facing adverse health conditions (prolonged hospitalization, periodic rehabilitation services, and low self-care ability), older adults may also fall subject to unmet healthcare needs more than any other age group [20].

Yahyavi Dizaj et al. (2020) mentioned medical care as the basic need of older adults, and they are also the most popular group to receive these services [21]. However, studies have found that socioeconomic factors affect long before the involvement of health care, among which place of residence plays a crucial health determinant for older adults [21]. Among the ageing population, the critical challenges to healthy ageing are poverty, nutrition, declining social support, and poor health services. Often, variation is observed by geographical region [22]. Additionally, this population group also suffers more from being the non-priority in the nutrition and health programs, as they primarily target children and women [23].

Additionally, in Asian societies, older adults have had more support from their family and kinship; however, the era of modernization caused the breakdown of the functional and structural form of the traditional family. These societal modifications have influenced the care and well-being of the ageing population [24]. Globally, rural-urban differences have been observed at various levels, including healthcare access and service utilization and the prevalence of health conditions [25]. Rural demographics have widely been associated with a lower level of education, income, and life expectancy than their urban counterparts have [26]. Similar trends have been identified among the ageing communities, as the rural dwellers reported lower health status and quality of life than the urban older adults [22, 25]. However, urban older adults suffered more from lifestyle-induced morbidities and depression than their urban counterparts did [16, 27, 28].

Malnutrition is a more significant concern as older adults consume less food with an increase in age; in such cases, compromising in quality and quantity of food may affect severely. Based on the literature, developing countries struggle to meet the required quality and quantity of food more than developed countries. However, due to the lack of data for older adults, the worse scenario of unmet need for food among older adults may be expected and needs to be explored. World Population and Ageing Report (2019) projected the share of the ageing population (65 and above) to double in size from 2019 to 2050, from which India will be home to the most significant number of older adults, increasing from 6 to 14% [4]. Tools like the Global Age Watch Index developed by HelpAge International have helped track older adults’ well-being, including four key areas: income security, health status, employment, and enabling environment [29, 30]. In the global ranking 2016, India ranked 71st out of 96 countries and performed worst in the health and economic security domain (Global Age Watch Index, 2015) [30].

Being a diverse country, the geographical location and cultural background present tribulations in achieving the growth and well-being of the senior population. For instance, the older section’s demand for food and healthcare is influenced by dietary practices, familial ties, and the stigma associated with dietary practices [31]. While in India, social welfare programmes, including pension plans, healthcare subsidies, and food assistance programmes, are not widespread or are not fully available to the elderly population as they are the dependent group and have little to no savings [32, 33]. These figures indicate the vulnerability of older adults and their subsequent health concerns. Recent development in geriatric research has focused on nutrition and healthcare utilization. However, there is a limited number of studies examining rural/urban differences at the national level. This study examines rural/urban differences in unmet needs for food and healthcare among Indian older adults.

## Method

### Data source and study population

The Longitudinal and Ageing Survey of India (LASI) is a comprehensive nationwide panel survey of India’s health, economics, and social drivers and implications of population ageing. It is a nationally representative survey of 72,250 seniors aged 45 and over from all Indian states and union territories. It is the largest and the first longitudinal ageing study in India.

**Fig. 1:**
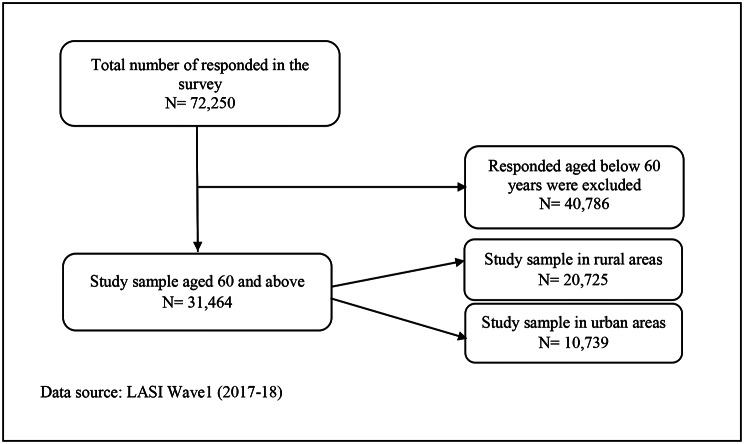
Sampling selection process for analysis in the study using LASI wave 1, 2017-18. Data source: LASI Wave1 (2017-18)

LASI followed a multistage stratified area probability cluster sampling design. For each state, a three-stage sampling design in rural areas and a four-stage sampling design in urban areas were followed. Primary Sampling Units (PSUs), or sub-districts (Tehsils/Talukas), were chosen in each state/UT in the first stage, and at the second stage, wards in urban areas and villages in rural areas were selected in the PSUs. In rural areas, households were selected from selected villages in the third stage. However, sampling in urban areas involved an additional stage. Specifically, one Census Enumeration Block (CEB) was randomly selected in the third stage in each urban area. In the fourth stage, households were selected from the selected CEB. The household was randomly selected, and the information was collected from one or more knowledgeable adults in the HH. As the study only focused on older adults, 60 years and above samples were (N = 31,464) included after excluding samples with missing information. The study population selection process was further elaborated in Fig. 1.

### Outcome variables

The outcome variables for this study are the unmet need for health care and the survey’s unmet need for food. The older adults who reported “poor and very poor perceived health” and “did not visit any healthcare facility in the past 365 days” were considered to estimate the unmet need for healthcare. A dichotomous variable was created. The older adults who suffered from unmet health needs were coded as “yes” or “no”. The unmet need for food among older adults has been taken from the direct question asked in the survey “in the last 12 months, did you ever reduce the size of your meals or skip meals because there was not enough food in your household?“; frequencies of the respondents were provided in “yes” or “no”.

### Predictor variables

A set of demographic, social, and economic factors were used to show their association with the unmet need for health and food. The age of the respondents was coded into broad age groups as 60 to 74 years, 74 to 84, and 85 and above. The other factors include sex categorised as male and female. The religion of the older adults was considered and categorized as Hindu, Muslim, Christian, and Others. The social group was considered categorized as Scheduled Castes (ST), Scheduled Tribes (SC), Other Backward Classes (OBC), and Others. The SC, ST and OBC were the disadvantageous groups performing poorly on different social and economic developmental indicators. Monthly Per Capita Consumption Expenditure (MPCE) was considered, and the sample was categorized into rich, middle, and poor. Living arrangement of the elderly was considered and the sub-categories were; residing alone, residing with spouse and children, residing with spouse/children/others, and residing with others. The inclusion of education level of the older adults were used as No education, less than primary level education, primary level education, and secondary and above. The older adults’ marital status was considered a predictor variable with categories of currently in union and currently not in a union. Another predictor variable in this study was the household size of the respondents, with a household size of 1 to 5 members and a household size of six or more members.

### Statistical analysis

The study variables were first described using descriptive statistics. Bivariate analysis was done to show the prevalence of unmet health and food needs among older adults across the selected covariates. The bivariate analysis also employed chi-square test to assess the significance of the difference, and the results were shown using the p-value. Logistic regression was used to show the association between the unmet need for food and health and the place of residence. Adjusted and unadjusted models were prepared to show the effect of place of residence on unmet needs for food and health among older adults. The results of the regression were presented in Odds Ratio (OR) and 95% confidence interval.

Further, decomposition analysis was applied to assess the contributing factors towards the rural-urban gap in unmet need for health care and food. The sampling weights provided by LASI were used in the analysis. All analyses were carried out using STATA version 14.

## Results

The socioeconomic and demographic characteristics of the older adults are summarised by place of residence in Table 1. The proportion of older adults was almost equally distributed in both urban and rural areas. Females were more than males. However, in urban areas, the proportion of females was around 10% more than their male counterparts. In comparison, in rural areas, the difference was only 4%. There was a notable difference in the proportion of social groups; among rural dwellers, the proportion of Scheduled Castes was 22%, and it was 11% in urban areas. Considering educational attainment, the highest proportion of older adults were illiterate. However, the proportion in urban areas was 34%, and in rural areas, it was 66%. Among urban dwellers, older adults, 16% were highly educated; contrastingly, it is only 3% among the older adults living in rural areas. Around 6% of rural older adults and around 4% of urban older adults have reported living alone. Considering marital status, in both rural and urban areas, the highest proportion of older adults was currently in the union, although the proportion not in the union was dominated by urban older adults.

**Table 1:** Background characteristics of elderly by place of residence in India, LASI Wave-1 (2017-18)

Variables	Urban (%)	N	Rural (%)	N
Age				
60–74	77.61	8,500	77.11	16,215
75–84	18.04	1,813	17.91	3,488
85+	4.35	426	4.98	1,022
Sex				
Male	45.02	5,021	48.47	10,077
Female	54.98	5,718	51.53	10,648
Religion				
Hindu	79.13	7,624	83.52	15,413
Muslim	14.93	1,740	9.75	1,991
Christian	2.51	904	3.01	2,246
Others	3.43	471	3.72	1,075
Social Group				
SC	11.36	1,261	22.06	3,879
ST	3.02	1,139	10.25	4,034
OBC	47.08	4,046	44.46	7,840
Others	38.54	4,293	23.23	4,972
MPCE				
Poor	42.77	4,483	43.69	8,478
Middle	20.55	2,154	21.11	4,262
Rich	36.68	4,102	35.21	7,985
Education				
No education	34.05	3,696	65.9	13,193
Less than primary	11.92	1,364	11.23	2,417
Primary	14.07	1,559	9.98	2,220
Secondary	24.19	2,406	9.59	2,208
Higher secondary and	15.77	1,714	3.29	687
Living Arrangement				
alone	4.13	468	6.33	1,154
living with spouse an	40.69	4,673	40.59	8,792
spouse/children/other	50.17	4,982	47.05	9,651
with others only	5.02	616	6.04	1,128
Marital Status				
currently married	58.8	6,650	62.81	13,270
Not in union	41.2	4,089	37.19	7,455
Household size				
6+	40.74	4,043	41.21	8,532
1–5	59.26	6,696	58.79	12,193

Note: LASI provided sampling weights were applied

**Fig. 2:**
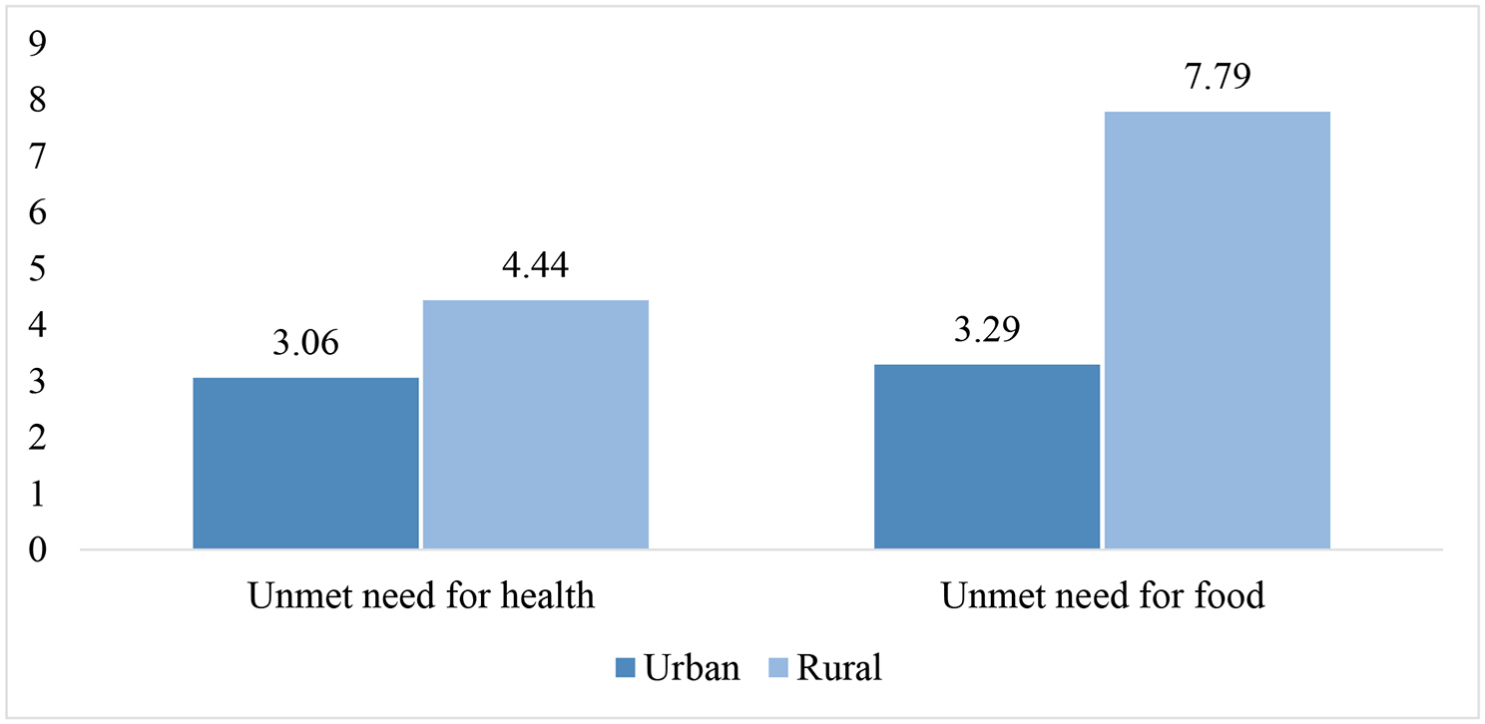
Unmet need for health and food among the elderly in India by place of residence, evidence from LASI Wave 1, 2017-18

Figure 2 shows the prevalence of unmet needs for food and health among older adults aged 60 and above by their place of residence. Rural older adults were more vulnerable to both deprivations than their urban counterparts were. Around 4% of rural older adults and 3% of urban older adults had reduced the size of their meals or skipped meals. In comparison, the proportion of older adults having poor perceived health and not visiting any health facility was 8% in rural areas and 3% in urban areas.

The variation in the prevalence of unmet needs for food by the socioeconomic and demographic characteristics of older adults is shown in Table 2. A significant urban-rual difference in unmet need for food was observed for the selected characteristics. Female older adults had experienced more unmet needs than male older adults had. However, female older adults in rural areas were the most vulnerable (8%). Irrespective of the place of residence, the prevalence of unmet need for food was highest among Muslims, although Muslim Older adults residing in rural areas experienced around 5% more unmet needs than their urban counterparts did. Among the rural dwellers, respondents belonging to SC (11%) community had the highest unmet need, while in urban areas, the most vulnerable community was ST (9%). The individual with high Monthly Per Capita Expenditure (MPCE) had a lesser unmet need for food and vice-versa. However, the rich respondents from rural areas (7%) experienced more unmet needs for food than the poor respondents from urban areas (4%) did. Older adults with no education or less education suffered from higher unmet needs for food than the educated; in particular, older adults with less than primary education reported unmet needs of 5% in urban areas and 10% in rural areas. The living arrangement was being critical indicator well-being of the older adults; those who are residing alone had suffered the most in meeting the need for food, in urban areas at 11% and in rural areas at 16% respectively. Considering marital status, older adults currently not in the marital union were more vulnerable in meeting food needs than the older adults currently married. In urban and rural areas, the unmet need for food among the older adults not in the union was 4% and 9%, respectively. However, the currently married older adults of rural areas (7%) were more vulnerable than the urban older adults who were not in the union. Individuals from bigger households (6 or more household members) had a higher unmet need for food than those from smaller households (1 to 5 household members).

**Table 2:** Unmet need for food among elderly by place of residence in India, LASI Wave-1 (2017-18)

Variables	Urban	Rural	p-value
Age			
60–74	3.21	7.74	0.000
75–84	2.67	7.86	0.000
85+	4.22	7.23	0.018
Sex			
Male	2.86	7.53	0.000
Female	3.4	7.92	0.000
Religion			
Hindu	2.94	7.32	0.000
Muslim	4.58	11.1	0.001
Christian	3.49	10.17	0.015
Others	2.35	6.29	0.000
Social Group			
SC	4.76	10.7	0.000
ST	9.15	8.72	0.002
OBC	2.98	6.58	0.000
Others	2.44	6.7	0.000
MPCE			
Poor	3.99	9.03	0.000
Middle	3.46	6.66	0.000
Rich	2.04	6.76	0.000
Education			
No education	4.9	8.3	0.001
Less than primary	4.97	9.9	0.000
Primary	2.48	5.67	0.023
Secondary	1.47	5.51	0.002
Higher secondary and	1.09	1.71	0.325
Living Arrangement			
alone	10.62	15.79	0.149
living with spouse an	1.93	6.07	0.000
spouse/children/other	3.3	7.75	0.000
with others only	6.08	10.48	0.069
Marital Status			
currently married	2.54	7.03	0.000
Not in union	4.02	8.92	0.000
Household size			
6+	2.21	5.5	0.000
1–5	3.79	9.3	0.000

Note: LASI provided sampling weights were applied

Table 3 exhibits the differential in unmet needs for healthcare among older adults across the characteristics. The chi-square test showed a highly significant rural-urban differential in unmet needs for healthcare for majority of characteristics. Reporting of unmet needs had increased with age; the oldest olds aged 85 or older had reported 7% and 11% in urban and rural areas, respectively. Irrespective of their place of residence, females were subject to higher unmet needs than their male counterparts. However, the rural males (4%) were almost equally vulnerable as the urban females (4%). Christian older adults reported the highest unmet need in both urban (10%) and rural (9%) areas, whereas Muslim individuals have been identified to have the least unmet need (U- 2%; R- 2%). In urban areas, OBCs had a higher vulnerability in meeting health needs (4%); in contrast, in rural areas, ST individuals showed the highest unmet need (6%). Moreover, older adults in the middle MPCE had the least unmet need in urban (2%) and rural (3%) areas, respectively. Individuals with the highest educational level reported less unmet health needs than those with less education. Mainly, the unmet need reporting was 2% among urban older adults and rural older adults with a secondary and higher level of education. The older adults residing with others or alone were subject to a higher unmet need for health than those residing with their kin. For instance, individuals habituating with others had reported unmet needs 5% in urban areas and 9% in rural areas, respectively. Moreover, the older adults currently in the union were better off meeting health needs than those not in the union; In both urban and rural areas, older adults not in the union reported 4% and 6% unmet needs, respectively.

**Table 3:** Unmet need for healthcare among older adults by place of residence in India, LASI Wave-1 (2017-18)

Variables	Urban	Rural	p-value
Age			
60–74	9.67	6.88	0.000
75–84	9.1	7.22	0.000
85+	15.45	6.59	0.025
Sex			
Male	10.08	6.45	0.000
Female	9.61	7.37	0.000
Religion			
Hindu	10.24	7.17	0.000
Muslim	6.84	4.04	0.000
Christian	14.8	11.8	0.000
Others	9.66	5.05	0.001
Social Group			
SC	8.89	7.86	0.000
ST	12.41	7.76	0.000
OBC	9.65	6.66	0.000
Others	10.11	6.16	0.000
MPCE			
Poor	10.36	7.26	0.000
Middle	10.6	6.38	0.001
Rich	8.76	6.84	0.000
Education			
No education	8.99	6.51	0.000
Less than primary	7.74	7.65	0.145
Primary	9.51	7.45	0.000
Secondary	8.27	7.92	0.004
Higher secondary and	15.87	8.24	0.000
Living Arrangement			
alone	7.82	6.77	0.128
living with spouse an	10.2	6.62	0.000
spouse/children/other	9.73	6.89	0.000
with others only	9.38	9.39	0.919
Marital Status			
currently married	9.66	6.85	0.000
Not in union	10.06	7.04	0.000
Household size			
6+	7.78	6.8	0.000
1–5	11.23	7.01	0.000

The results of multivariate logistic regression are shown in Table 4. The link between unmet need for food and unmet need for health with a place of residence has been shown differently in the adjusted and unadjusted models. Unadjusted for other covariates, place of residence had shown significant association with the unmet need for food as well as unmet need for health. In particular, rural older adults were 86% (OR = 1.86; 95% CI = 1.65–2.09) more likely to suffer from an unmet need for food than urban older adults were. Moreover, the unmet need for health was 17% (OR = 1.17; CI = 1.04–1.33) more likely to occur among rural respondents than urban respondents. While adjusting for predictor variables, the likelihood of suffering from an unmet need for food among rural older adults was significantly higher by 53% (OR = 1.53; CI = 1.35–1.74) from urban older adults. However, place of residence did not have a significant association with unmet needs for health in the adjusted model. The age of the respondents had a significant association with their health needs; for instance, older adults aged 75–84 were 68% more likely to incur unmet needs, while for the age group 85 and above, the odds ratio was 3.36 (OR = 3.36; CI 95% =2.79–4.04). Sex of the individuals was significantly associated with both the unmet need for food and health; in particular, females were 11% (OR = 0.89; CI 95% =0.79-1.00) less likely to suffer unmet need for food and 14% (OR = 1.14%; CI 95% =1-1.3) more likely to report unmet need for health than males. Social groups were statistically associated with an unmet need for food. The OR for ST, OBC, and others compared to the reference category of SC was 0.74 (CI 95% =0.62–0.87), 0.73 (CI 95% =0.64–0.84), and 0.62 (CI 95% =0.53–0.73), respectively. Moving up in the MPCE decreases the likelihood of incurring unmet needs, as rich subjects were 43% (OR = 0.57; CI 95%=0.51–0.65) less likely to report unmet needs for food and 41% (OR = 0.59; CI 95%=0.51–0.68) were less likely to report unmet need for health than the poor individuals were. Compared to the older adults living with spouses or children or others, the older adults living alone had 2.55 odds (OR = 2.55; CI 95%= 2.12–3.06) of suffering from an unmet need for food. Moreover, compared to currently married older adults, their counterparts were 19% (OR = 0.81; CI 95%= 0.7–0.95) less likely to report unmet need for food and 22% (OR = 1.22; CI 95% =1.02–1.46) more likelihood of reporting the unmet need for health. The number of members in a household of the respondents had a significant impact on meeting their need for food and health; for instance, the older adults belonging to smaller households were more vulnerable and had reported 55% (OR = 1.55; CI 95% =1.37–1.75) more unmet need for food and 13% (OR = 1.13; CI 95% =0.99–1.29) more unmet need for health than the reference category.

**Table 4:** Association between the unmet need for food and health with socio-economic characteristics of elderly in India, LASI Wave-1 (2017-18)

Variables	Unmet need for food		Unmet need for health	
	Unadjusted	Adjusted	Unadjusted	Adjusted
Residence				
Urban	1.00	1.00	1.00	1.00
Rural	1.86***(1.65 2.09)	1.53***(1.35 1.74)	1.17**(1.04 1.33)	1.05 (0.91 1.2)
Age				
60–74		1.00		1.00
75–84		0.98 (0.85 1.11)		1.68***(1.46 1.94)
85+		0.94 (0.74 1.19)		3.36***(2.79 4.04)
Sex				
Male		1.00		1.00
Female		0.89**(0.79 1)		1.14*(1 1.3)
Religion				
Hindu		1.00		1.00
Muslim		1.53***(1.32 1.78)		0.71***(0.57 0.88)
Christian		0.79**(0.64 0.98)		1.79***(1.47 2.17)
Others		1.13 (0.89 1.43)		0.62***(0.44 0.89)
Social Group				
SC		1.00		1.00
ST		0.74***(0.62 0.87)		0.93 (0.75 1.15)
OBC		0.73***(0.64 0.84)		1.1 (0.93 1.31)
Others		0.62***(0.53 0.73)		0.88 (0.72 1.07)
MPCE				
Poor		1.00		1.00
Middle		0.74***(0.65 0.85)		0.69***(0.59 0.8)
Rich		0.57***(0.51 0.65)		0.59***(0.51 0.68)
Education				
No education		1.00		1.00
Less than primary		1.02 (0.87 1.19)		0.91 (0.76 1.1)
Primary		0.66***(0.55 0.8)		0.9 (0.74 1.1)
Secondary		0.52***(0.42 0.63)		0.84*(0.68 1.03)
Higher secondary and		0.33***(0.23 0.47)		0.67**(0.49 0.93)
Living Arrangement				
living with spouse/children/other		1.00		1.00
living alone		2.55***(2.12 3.06)		1.05 (0.83 1.34)
living with spouse and children		0.75***(0.65 0.87)		0.85*(0.71 1.02)
living with others only		1.4***(1.13 1.73)		1.21*(0.98 1.5)
Marital Status				
currently married		1.00		1.00
Not in union		0.81***(0.7 0.95)		1.22**(1.02 1.46)
Household size				
6+		1.00		1.00
1–5		1.55***(1.37 1.75)		1.13*(0.99 1.29)

*Significant at 10% level. **Significant at 5% level. ***Significant at 1% level

The rural and urban disparity in experiencing unmet needs has been established in the previous results, which indicates the need to decompose the unmet needs of residents and identify the contributing factors to the gap. The factors that contributed majorly to the difference in unmet need for food were education (34.98%), social group (6.58%), living arrangements (3.34%) and MPCE quintile (2.84%). Similarly, for the unmet need for health, the factors that contribute the most to the rural-urban gap are education (28.2%), household size (2.32%), and MPCE quintile (1.27%) (Table 5).

**Table 5:** Decomposition analysis to show the percent contribution towards the rural-urban differential in the unmet needs

Characteristics	Unmet need for Food		Unmet need for Health	
	Coefficient	Percentage contribution (%)	Coefficient	Percentage contribution (%)
Sex	-0.00027	0.951	-0.0001	-0.247
Education	-0.0099***	**34.986**	0.01091***	**28.203**
Religion	0.00001	-0.048	-0.00026**	-0.684
Social Group	-0.00186*	**6.583**	-0.00255**	-6.594
MPCE quintile	-0.0008**	**2.844**	0.00049**	**1.270**
Living arrangement	-0.00094**	**3.338**	0.00014	0.360
Marital status	0.00158***	-5.600	-0.00003	-0.074
Household size	0.00261***	-9.241	0.0009**	**2.319**
**Total**		**33.935**		**24.567**

*Significant at 10% level. **Significant at 5% level. ***Significant at 1% level

## Discussion

The ageing process is accompanied by frailties and poor health conditions, which makes it important to cater to their basic health needs. The present study explores the rural-urban differentials in the unmet need for food and health among the older adult population in India. The findings show the vulnerability of the older adults’ rural residents in terms of the basic needs of health and diet. The proportion of unmet needs was significantly higher in rural areas than in urban areas in terms of both food and health. Generally, the older adult population is most vulnerable regarding restrictions on healthcare access and burden [34]. The deprived older adults in rural areas have further aggravated vulnerable conditions. A field-based study had shown that ‘poor quality of services, ‘lack of control over scheduling’ and ‘system level inflexibility’, were some of the frustrations reported by rural elderlies regarding health care services [35]. The supportive services for older adults in rural areas lack availability, basic understating of the patient’s needs and inability to meet their needs [36].

The risk of unmet need for food among rural residents was a lot higher than the urban counterpart. Many meal services that promote health, nutrition, and independent living of frail elderlies in both rural and urban areas are an essential component of ageing support systems [37]. The experiences of the older adults due to the differences in residences also contribute to the unmet needs. Rural counterparts may face problems in availing enough food and have an increased risk of poor nutritional intake, poor health, and paying for basic needs like housing and medications [38–40].

The socioeconomic and demographic factors were also explored as covariates determining the unmet need. Among older adults also, the older ones are more vulnerable, thus showing that increasing age is a significant predictor [41]. The results showed that female older adults were more prone to experience unmet needs than males irrespective of residence [42, 43]. However, rural males were also quite vulnerable in this regard. This finding is in line with a study by Momtaz et al. (2012)where gender is a significant predictor of unmet needs of rural residents [44], which showed that older men in rural areas have a higher risk of facing the unmet need for social care [45] The economic condition did show significant relation overall where the poorer were more vulnerable [46]. Studies have shown that low-income families have a higher risk of having unmet needs among older adults, but the richer in the rural areas were also likely to face the unmet need for food and health, indicating the residential vulnerability [43]. The study findings also show that lesser educated older adults faced more unmet needs of health than higher educated ones [47].

Living arrangements emerged as a significant predictor, indicating that older adults living alone were more likely to face the unmet need for food [40, 45]. A study by Singh et al. (2016) also showed a similar finding where older adults living alone have higher odds of facing unmet needs in physical activities, including eating [48]. Also, older adults who were unmarried or currently not in the union were more vulnerable to having an unmet need for food [45]. Thus, indicating that loneliness contributes to the inability to meet basic food needs. Again, if the household size is too large in number, then also the matter of unmet needs arises. When the household has six or more members, it becomes difficult to meet basic food needs [49].

The study contributes significantly to the literature and has few key messages. Firstly, a considerable proportion of older adults in India are experiencing unmet need of basic food and health. Secondly, the socioeconomic and demographic characteristics have shown economic and residential vulnerability does factor into the scenario of basic unmet needs. Thirdly, the factor of loneliness has also shown that it leads to difficulty in meeting food needs. Fourthly, the decomposition analysis to show the contributing factors towards the rural-urban gap in unmet needs is a methodological contribution as it has not been used in this regard. The decomposition results show that education, economic condition and household size are major attributable factors. These key results can be taken to point towards an evidenced link between the perceived health status of the older individuals and the risk of experiencing unmet health and food needs through the linkage of socioeconomic and demographic vulnerability. These linkages transcend the context of country-specific policies that are needed to highlight the significance of safeguarding the well-being of the older adult population.

Though, the study has strengths in terms of strong methodology and robust estimates, there are some limitations. The first limitation can be of the indicator for unmet need, which still can have more scope of refinement. Further, the analysis can be subject to potential heterogeneity due to the factors not considered in the study, such as financial dependence or diagnosed conditions. Studies based on national surveys are always subject to recall biases and limitations of cross-sectional nature. The experience of unmet need can be dynamic, and inferring from a one-point national level data may not capture the entire scenario. It can be recommended to investigate over time in this regard considering more intricate details.

In conclusion, the present study has highlighted that a significant proportion of older adults still cannot meet their basic needs of health and food. Some of the identified attributable factors are the economically backward, lesser educated, residing in rural areas, living alone and having a large household size. This indicates towards making targeted policy-level efforts considering the economic and residential vulnerability identified in the study. There is a need for primary care services that can provide targeted help to older adults in rural communities. The study findings can help generate a plan for providing quality healthcare and food security services to the economically vulnerable, rural elderlies and the ones who need the most.

## Data Availability

The study is based on the secondary data source available in the public domain. For the data, please follow the link www.iipsindia.ac.in/lasi.

## References

[1] Waite LJ (2004). Aging, health, and public policy: demographic and economic perspectives.

[2] Partridge L, Deelen J, Slagboom PE. Facing up to the global challenges of ageing. Nat 2018 Sep;561(7721):45–56. 10.1038/s41586-018-0457-8 30185958

[3] Raju SS. Studies on ageing in India: a review. Popul Ageing India. 2014 Jul 14;180.

[4] World Population Ageing (WPA). (2019). United Nations, Department of Economic and Social Affairs (DESA). https://www.un.org/en/development/desa/population/publications/pdf/ageing/WorldPopulationAgeing2019-Highlights.pdf [Accessed on 27 May 2022].

[5] World Health Organisation (WHO). Ageing and Health. (2021) https://www.who.int/news-room/fact-sheets/detail/ageing-and-health [Accessed on 28 May, 2022].

[6] Mitchell E, Walker R. Global ageing: successes, challenges and opportunities. Br J Hosp Med 2020 Feb 2;81(2):1–9. 10.12968/hmed.2019.0377 32097073

[7] World Health Organisation (WHO). Global Health Observatory data repository. World Health Statistics. (2020) https://apps.who.int/gho/data/view.main.SDG2016LEXREGv?lang=en [Accessed on 28 May, 2022].

[8] Feng Z, Phillips DR. Geographical gerontology. InEncyclopedia of Gerontology and Population Aging 2022 May 24 (pp. 2078–92). Cham: Springer International Publishing.

[9] Hazra NC, Rudisill C, Gulliford MC. Determinants of health care costs in the senior elderly: age, comorbidity, impairment, or proximity to death? Eur J Health Econ. 2018 Jul;19(6):831–42. 10.1007/s10198-017-0926-2 PMC6008359 28856487

[10] de Morais C, Afonso C, De Almeida MD. Ageing and food consumption in Portugal: new or old paradigms?. Br Food J. 2010 May 18.

[11] Brown JE, Isaacs JS, Krinke UB. Nutrición en las diferentes etapas de la vida.

[12] Wolfe WS, Olson CM, Kendall A, Frongillo EA Jr. Understanding food insecurity in the elderly: a conceptual framework. J Nutr Educ. 1996 Mar;28(1):92–100.

[13] Wolfe WS, Olson CM, Kendall A, Frongillo EA Jr. Hunger and food insecurity in the elderly: its nature and measurement. J Aging Health. 1998 Aug;10(3):327–50. 10.1177/089826439801000304 10342935

[14] Fernandes SG, Rodrigues AM, Nunes C, Santos O, Gregório MJ, De Sousa RD, Canhão H (2018). Food insecurity in older adults: results from the epidemiology of chronic diseases cohort study 3. Front Med.

[15] Feeding America Organisation. Hunger in America. Senior Hunger. Accessed using: https://www.feedingamerica.org/hunger-in-america/senior-hunger-facts (Accessed on: 15-07-2022).

[16] Liu YH, Chang HJ, Huang CC. The unmet activities of daily living (ADL) needs of dependent elders and their related factors: an approach from both an individual-and area-level perspective. International Journal of Gerontology. 2012 Sep 1;6(3):163-8.

[17] Alonso J, Orfila F, Ruigomez A, Ferrer M, Antó JM (1997). Unmet health care needs and mortality among spanish elderly. Am J Public Health.

[18] Park S, Kim B, Kim S. Poverty and working status in changes of unmet health care need in old age. Health Policy. 2016 Jun 1;120(6):638–45. 10.1016/j.healthpol.2016.03.004 27025977

[19] World Health Organisation (WHO). Ageing and Health. (2021) https://www.who.int/news-room/fact-sheets/detail/ageing-and-health [Accessed on 28 May, 2022].

[20] Jenson J. Health Care spending and the aging of the population.

[21] Yahyavi Dizaj J, Tajvar M, Mohammadzadeh Y. The effect of the presence of an elderly member on health care costs of iranian households. Iran J Ageing 2020 Feb 10;14(4):462–77.

[22] Chen SH, Cheng HY, Chuang YH, Shao JH. Nutritional status and its health-related factors among older adults in rural and urban areas. J Adv Nurs. 2015 Jan;71(1):42–53. 10.1111/jan.12462 24894954

[23] Sciubba JD. Explaining campaign timing and support for a UN Convention on the rights of older people. Int J Hum Rights 2014 Jul 4;18(4–5):462–78.

[24] Kumar R. Social determinants of health among elderly: an anthropological study. Int J Res Sociol Social Anthropol. 2013 Aug;15(1):11–6.

[25] Phaswana-Mafuya N, Peltzer K, Pengpid S. Rural–urban health disparities among older adults in South Africa. African Journal of Primary Health Care and Family Medicine. 2019 Jan 1;11(1):1–6. 10.4102/phcfm.v11i1.1890 PMC6620551 31296012

[26] Dong X, Simon MA. Health and aging in a chinese population: urban and rural disparities. Geriatr Gerontol Int. 2010 Jan;10(1):85–93. 10.1111/j.1447-0594.2009.00563.x 20102387

[27] Goeres LM, Gille A, Furuno JP, Erten-Lyons D, Hartung DM, Calvert JF, Ahmed SM, Lee DS. Rural‐urban differences in chronic disease and drug utilization in older oregonians. J Rural Health. 2016 Jun;32(3):269–79. 10.1111/jrh.12153 PMC5116378 26515108

[28] Abalu O, Segura L, Dávila MG, Abuawad A. Rural–Urban Health Disparities Among Older Adults in South Africa. American Journal of Public Health. 2020 Aug 1;110(8):1110-.

[29] Brenes-Camacho G. Favourable changes in economic well-being and self-rated health among the elderly. Social science & medicine. 2011 Apr 1;72(8):1228-35. 10.1016/j.socscimed.2011.02.027 PMC3084457 21440352

[30] Global Age Watch Index. (2015). Help Age International. Accessed (12/06/22) using: https://www.helpage.org/global-agewatch/population-ageing-data/country-ageing-data/?country=India.

[31] Pérez-Zepeda MU, Castrejón-Pérez RC, Wynne-Bannister E, García-Peña C (2016). Frailty and food insecurity in older adults. Public Health Nutr.

[32] Alam M, James KS, Gridhar G, Sathyanarayana KM, Kumar S, Raju SS, Syamala TS, Subaiya L, Bansod DW. Report on the status of elderly in select states of India, 2011. New Delhi: United Nations Population Fund. 2012 Sep 4.

[33] Giridhar G, Subaiya L, Verma S. Older women in India: Economic, social and health concerns. Increased Awarenes, Access and Quality of Elderly Services. BKPAI (Building Knowledge Base on Ageing in India), Thematic Paper. 2015;2.

[34] Krůtilová V. Unmet need for health care–a serious issue for european elderly?. Procedia-Social and Behavioral Sciences 2016 May 31;220:217–25.

[35] MaloneBeach EE, Zarit SH, Spore DL. Caregivers’ perceptions of case management and community-based services: barriers to service use. J Appl Gerontol. 1992 Jun;11(2):146–59. 10.1177/073346489201100202 10171017

[36] Thomas CP, Payne SM. Home alone: unmet need for formal support services among home health clients. Home Health Care Serv Q. 1998 Oct;5(2):1–20. 10.1300/J027v17n02_01 10186163

[37] Li H. Unmet needs for supportive services: a comparison of rural and urban older adults. J social service Res 2006 Jul 26;32(3):19–39.

[38] Bengle R, Sinnett S, Johnson T, Johnson MA, Brown A, Lee JS. Food insecurity is associated with cost-related medication non-adherence in community-dwelling, low-income older adults in Georgia. J Nutr Elder 2010 May 17;29(2):170–91. 10.1080/01639361003772400 20473811

[39] Kushel MB, Gupta R, Gee L, Haas JS. Housing instability and food insecurity as barriers to health care among low-income Americans. J Gen Intern Med. 2006 Jan;21(1):71–7. 10.1111/j.1525-1497.2005.00278.x PMC1484604 16423128

[40] Ziliak JP, Haist M, Gundersen C. The causes, consequences, and future of senior hunger in America. Alexandria, VA: National Foundation to End Senior Hunger; 2008 Mar.

[41] Elango S. A study of health and health related social problems in the geriatric population in a rural area of Tamil Nadu. Indian J public health 1998 Jan 1;42(1):7–8. 10389498

[42] Allen SM, Mor V. The prevalence and consequences of unmet need: contrasts between older and younger adults with disability. Med Care. 1997 Nov;1:1132–48. 10.1097/00005650-199711000-00005 9366892

[43] Goel PK, Garg SK, Singh JV, Bhatnagar M, Chopra H, Bajpai SK. Unmet needs of the elderly in a rural population of Meerut. Indian Journal of Community Medicine. 2003 Oct 1;28(4):165.

[44] Momtaz YA, Hamid TA, Ibrahim R. Unmet needs among disabled elderly Malaysians. Social science & medicine. 2012 Sep 1;75(5):859 – 63. 10.1016/j.socscimed.2012.03.047 22632847

[45] Vlachantoni A. Unmet need for social care among older people. Ageing Soc. 2019 Apr;39(4):657–84.

[46] Lee JS, Fischer JG, Johnson MA. Food insecurity, food and nutrition programs, and aging: experiences from Georgia. J Nutr Elder 2010 May 17;29(2):116–49. 10.1080/01639366.2010.480895 20473809

[47] Srivastava AK, Kandpal SD. Social problems and basic unmet need of the elderly: a cross sectional study in rural field practice area of medical college, Dehradun. Indian J Community Health 2013 Sep 30;25(3):221–5.

[48] Singh A, Bairwa M, Goel S, Bypareddy R, Mithra P. Prevalence and predictors of unmet needs among the elderly residents of the rural field practice area of a tertiary care centre from Northern India. Malaysian J Med sciences: MJMS. 2016 Sep;23(5):44. 10.21315/mjms2016.23.5.6 PMC5101985 27904424

[49] Owoo NS. Demographic considerations and food security in Nigeria. J Soc Econ Dev. 2021 Jun;23(1):1.

